# Short‐term calorie restriction ameliorates genomewide, age‐related alterations in DNA methylation

**DOI:** 10.1111/acel.12513

**Published:** 2016-08-25

**Authors:** Chul Hong Kim, Eun Kyeong Lee, Yeon Ja Choi, Hye Jin An, Hyeong Oh Jeong, Daeui Park, Byoung Chul Kim, Byung Pal Yu, Jong Bhak, Hae Yung Chung

**Affiliations:** ^1^ Genomictree Inc. Daejeon Korea; ^2^ Molecular Inflammation Research Center for Aging Intervention (MRCA) College of Pharmacy Pusan National University Busan Republic of Korea; ^3^ Department of Predictive Toxicology Korea Institute of Toxicology Daejeon Korea; ^4^ Department of Physiology University of Texas Health Science Center San Antonio TX USA; ^5^ Biomedical Engineering Ulsan National Institute of Sciences and Technology Ulsan Korea

**Keywords:** aging, Bioinformatics, calorie restriction, DNA methylation, MeDIP‐Seq

## Abstract

DNA methylation plays major roles in many biological processes, including aging, carcinogenesis, and development. Analyses of DNA methylation using next‐generation sequencing offer a new way to profile and compare methylomes across the genome in the context of aging. We explored genomewide DNA methylation and the effects of short‐term calorie restriction (CR) on the methylome of aged rat kidney. Whole‐genome methylation of kidney in young (6 months old), old (25 months old), and OCR (old with 4‐week, short‐term CR) rats was analyzed by methylated DNA immunoprecipitation and next‐generation sequencing (MeDIP‐Seq). CpG islands and repetitive regions were hypomethylated, but 5′‐UTR, exon, and 3′‐UTR hypermethylated in old and OCR rats. The methylation in the promoter and intron regions was decreased in old rats, but increased in OCR rats. Pathway enrichment analysis showed that the hypermethylated promoters in old rats were associated with degenerative phenotypes such as cancer and diabetes. The hypomethylated promoters in old rats related significantly to the chemokine signaling pathway. However, the pathways significantly enriched in old rats were not observed from the differentially methylated promoters in OCR rats. Thus, these findings suggest that short‐term CR could partially ameliorate age‐related methylation changes in promoters in old rats. From the epigenomic data, we propose that the hypermethylation found in the promoter regions of disease‐related genes during aging may indicate increases in susceptibility to age‐related diseases. Therefore, the CR‐induced epigenetic changes that ameliorate age‐dependent aberrant methylation may be important to CR's health‐ and life‐prolonging effects.

## Introduction

Aging is a complex process that results in changes in the expression and regulation of various genes over time, which then leads to functional changes and the accumulation of cellular damage (Knapowski *et al*., 2002). In part, genetic background governs the aging and lifespan of an individual; however, environment and epigenetics also play major roles in determining physiological changes over a lifetime (Gartner, 1990; Gurland *et al*., 2004). During aging, a loss of DNA methylation occurs, as does hypermethylation in certain gene promoters (Wilson *et al*., 1987; Issa *et al*., 2001; Waki *et al*., 2003). However, the genomic distribution of methylation and the molecular mechanisms underlying methylome alterations in aging animals remain unclear.

DNA methylation, an essential epigenetic modification in mammals, occurs predominantly in the cytosine residue of CpG dinucleotides. This DNA methylation typically leads to transcriptional repression of adjacent genes. DNA methylation is essential for diverse biological processes such as development, X‐inactivation, imprinting, and aging (Johnson *et al*., 2012). Genomewide DNA hypomethylation plays an important role in genomic instability and carcinogenesis (Portela & Esteller, 2010). Methylation of promoter CpG islands (CGIs) is rare in normal tissues, but is frequent in cancer and is usually associated with transcriptional silencing of the gene (Jones & Baylin, 2007). The alteration of normal DNA methylation patterns is also linked to the aging process (Johnson *et al*., 2012).

Recently, an important technical advance for DNA methylation analysis has been reported. The method uses immunoprecipitation with an antibody against 5‐methylcytosine to enrich methylated DNA fragments (Jacinto *et al*., 2008). This methyl‐DNA immunoprecipitation (MeDIP)‐based approach enables the rapid identification of multiple CpG sites throughout the genome and can be combined with microarray analysis (MeDIP‐Chip). Furthermore, next‐generation sequencing (NGS) technology now offers robust, quantitative, and cost‐effective functional genomic strategies for analyzing methylated DNA regions. MeDIP in conjunction with NGS (MeDIP‐seq), therefore, provides a genomewide mapping technique that has been used successfully to profile global DNA methylation patterns in aging research (Salpea *et al*., 2012).

One of the most well‐known and potent modifiers of the aging process is the calorie restriction (CR) paradigm. CR is perhaps the most effective way known to extend maximum lifespan in many different species. In addition, CR is known to delay a wide range of aging‐associated diseases, such as cancer, diabetes, atherosclerosis, and cardiovascular and neurodegenerative diseases in mammals (Fontana & Klein, 2007). Even short‐term CR is shown to alter age‐dependent gene expression that results in phenotypic outcomes. As we previously reported, very short‐term (10‐day) CR attenuated age‐related inflammatory processes mediated by the oxidative stress‐related proteins NF‐κB and AP‐1 in aged rat kidney (Jung *et al*., 2009a). Furthermore, a recent study has shown that short‐term, 8‐week CR protected kidneys from renal senescence in aged rats though SIRT1 and AMPK activation (Ning *et al*., 2013). Thus, both long‐ and short‐term CR may affect the aging process by favorably influencing various aspects of human health. Finally, recent evidence suggests that changes in the DNA methylation status at specific gene loci can play essential roles in CR‐dependent lifespan extension (Munoz‐Najar & Sedivy, 2011).

In our study, we used NGS technology coupled with MeDIP to profile the kidney methylome of aged rats and examined the effect of short‐term CR on methylation. Here, we report age‐related aberrant methylation patterns at promoter regions and the association of these patterns with increased susceptibility to age‐related diseases. Using the methylation data, we could determine the effects of short‐term CR on the epigenetic modifications that occurred in the aged rats, which then could provide an explanation for the CR effects during aging.

## Results

### Generation and mapping of MeDIP DNA sequencing data

To investigate the methylome of aged rats, we conducted MeDIP‐Seq sequencing by comparing methylation patterns in young (6 months old), old (25 months old), and OCR (old with 4‐week, short‐term CR) rats. From the three experimental groups, 18 genomic DNAs (six rats per group) were prepared from rat kidney, and 18 MeDIP‐Seq libraries were generated from the enriched methylated fraction according to Illumina's sequencing protocol. We generated ~1.12 Gbp of clean sequence (22 million reads of 49 nt) from each MeDIP‐Seq library. The six sequence data from each experimental group were combined into one sequence set with 136 million reads. The pooled sequence data can minimize interindividual variation in the local DNA methylation patterns as reported to yield results comparable to those obtained from individual samples. The combined MeDIP‐Seq data of each group were mapped to the rat reference genome (UCSC rn5) with Bowtie2, and only unique alignments with MAQ ≥ 20 were considered for further bioinformatic analysis. The total mapping rate was ~96%, and the unique mapping rate (MAQ ≥ 20) was ~70% (Table 1), which indicated the high quality and low bias of our MeDIP‐Seq data (Table S1 and Fig. S1).

**Table 1:** Summary of sequencing and read mapping of MeDIP‐Seq data

Sample	Total clean reads (M)^a^	Data size (Gbp)	Q‐Score (Mean)	Mapped reads (M)^a^	Total mapping rate (%)	Unique mapped reads (M)^a^^,^ b	Unique mapping rate (%)^b^
Young	136.8	6.7	38.7	131.5	96	96.2	70.4
Old	137.2	6.7	38.5	132.1	96	95.6	69.9
OCR	136.3	6.7	38.7	131.3	96	96	69.7

a M; millions.

b Unique mapping means >= MAQ20 (mapping quality).

### Finding and identifying differentially methylation regions

In order to determine enriched DNA patterns of MeDIP relative to the CpG motif, the Bioconductor package, MEDIPS, was used to normalize and calculate RPKM and RMS (relative methylation score) values of the methylated regions from MeDIP‐Seq data as well as to identify differentially methylated regions (see Methods). First, we calculated the short‐read coverage (windows bin size and extend value = 500 bp) and obtained average RPKM and RMS values for young, old, and OCR rats.

Then, to find DMRs (differentially methylated regions), each methylation value in the 500‐bp regions was compared between young, old, and OCR (old with short‐term CR) rats, generating three DMR profiles for each comparison. Significant DMRs were filtered from the profiles using the cutoff criteria of Poisson test *P*‐value < 0.05 with methylation fold change ≥ 1.33 for hypermethylation, and ≤ 0.75 for hypomethylation (as the default of MEDIPS). Here, a significant DMR profile was defined as follows: the OLD profile from comparing old rats with young rats; the OCR profile from comparing OCR (old with short‐term CR) rats with young rats; and the OCR‐O profile from comparing OCR (old with short‐term CR) rats with old rats (a comparison design is provided in Fig. S2, and full DMR profiles with methylation fold changes, *P*‐value, and annotations from each comparison are listed in Tables S2, S3, and S4). From the comparison between old and young rats, we identified 119 070 DMRs (i.e., the OLD profile showed 53 323 hypermethylated and 65 747 hypomethylated regions), which suggests that aberrantly methylated regions accumulated during aging (Table 2 and Table S2). When comparing OCR rats with young rats, 125 615 DMRs were found (i.e., the OCR profile showed 65 873 hypermethylated and 59 742 hypomethylated regions), suggesting that methylated regions accumulated during aging even with short‐term CR (Table 2 and Table S3). When comparing OCR with old rats, we found 103 209 DMRs (i.e., the OCR‐O profile showed 60 316 hypermethylated and 42 893 hypomethylated regions), suggesting that methylated regions by short‐term CR (Table 2 and Table S4).

**Table 2:** Differentially methylated regions (500 bp length) with significance (*P*‐value < 0.05) from comparisons between groups

	OLD (old vs. young)	OCR (OCR vs. young)	OCR‐O (OCR vs. old)
Hypermethylated	53 323	65 873	60 316
≥ twofold	33 018	44 392	40 342
Hypomethylated	65 747	59 742	42 893
≤‐twofold	39 257	36 126	27 156
Total	119 070	125 615	103 209

### Short‐term CR‐mediated age‐related methylation

**Figure 1:**
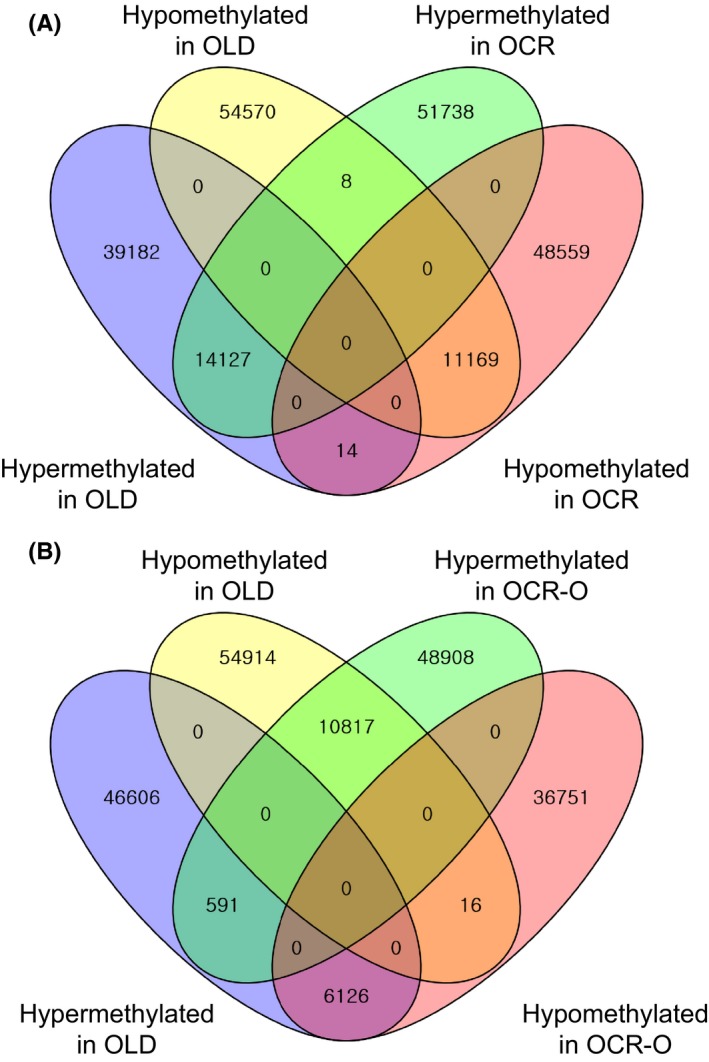
Venn diagram of significantly differentiated methylated regions between the OLD and OCR profiles (A), and the OLD and OCR‐O profiles (B) (please see Fig. S2 for the profile designation).

To determine ameliorated DMRs between the OLD, OCR, and OCR‐O profiles, we performed Venn diagram analyses for comparisons. Interestingly, comparisons between the OLD and OCR profiles show only 14 217 hypermethylated and 11 169 hypomethylated DMRs were shared among a hundred thousand of the OLD and OCR profiles (Fig. 1A). In contrast, when comparing the OLD and OCR‐O profiles, 591 hypermethylated and 16 hypomethylated regions were shared. Moreover, we observed that the methylation (6126 hypermethylated and 10 817 hypomethylated regions in the OLD profile) was reversed in the OCR‐O profile (Fig. 1B). These findings suggest that age‐related methylation might be ameliorated or reversed by short‐term CR. We found 85 gene promoters from the 6126 DMRs and 108 gene promoters from the 10 817 DMRs (Table S5). We performed pathway enrichment analysis using these promoters, but found no significantly enriched pathways. Nevertheless, these findings suggest that the promoters with ameliorated or reversed methylation by CR might be involved in changes of age‐related phenotypes.

### Methylation comparisons of CpG, repetitive elements, and gene body in DMRs

**Figure 2:**
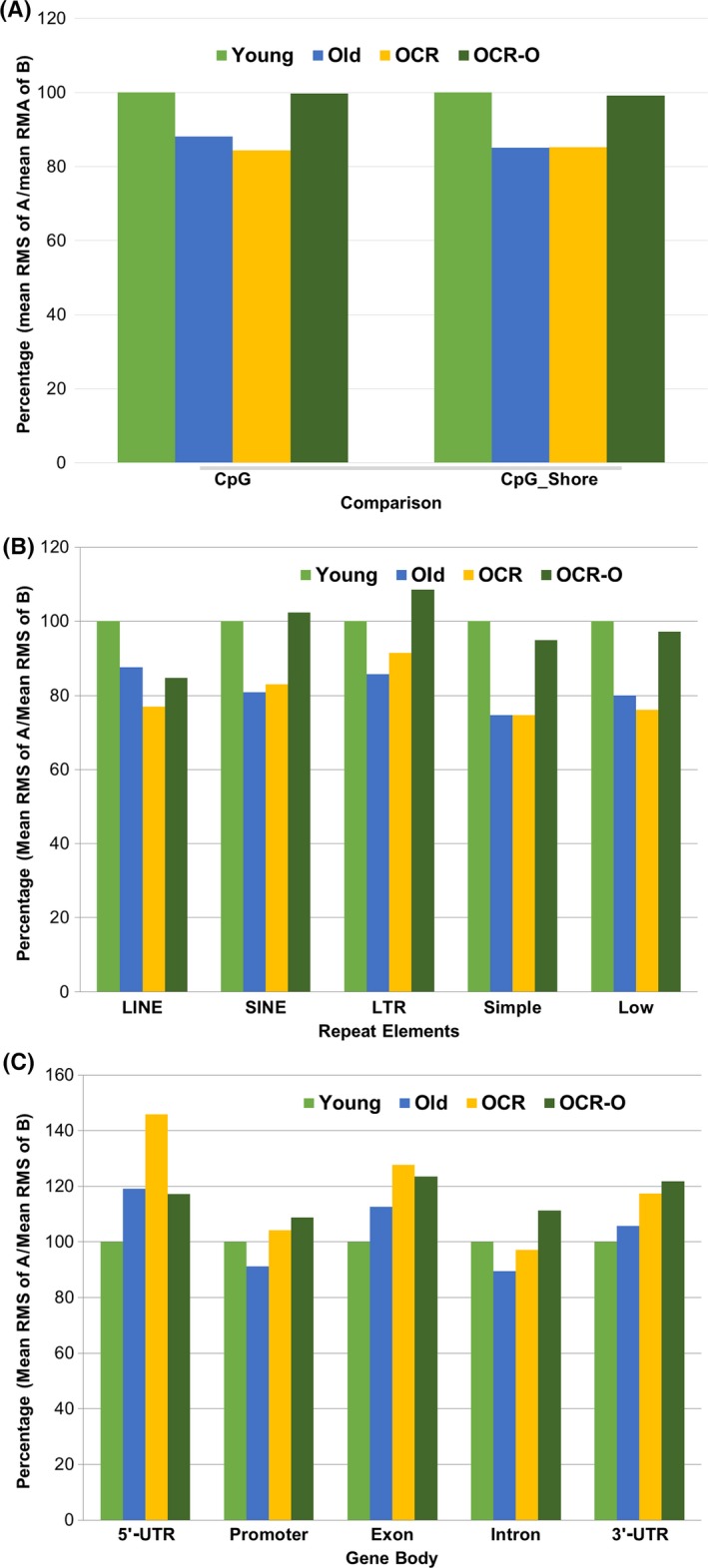
Methylation comparisons of differentially methylated regions in the OLD, OCR, and OCR‐O profiles. (A) CpG island and CpG shores, (B) interspersed repetitive elements, and (C) gene body regions. Bar means percentage of mean RMS of A/mean RMS of B. A or B indicates the regions in young, old, or OCR rats.

To assess methylation levels on CpG islands (CGI) and CpG shores (shore is defined as 2‐kb upstream and downstream of a CGI), we compared the mean RMS of CGI and CpG shores between the OLD, OCR, and OCR‐O profiles. Figure 2A shows that the methylation of CGI and CpG shores was decreased in OLD and OCR rats when compared with young rats. However, comparisons of methylation levels of CGI and CpG shores show no change between the OLD and OCR‐O profiles (Fig. 2A).

Interspersed repetitive sequences in the genome, such as long interspersed nuclear elements (LINE), short interspersed elements (SINE), and long terminal repeats (LTR), are generally methylated in normal tissues (Zhu *et al*., 2011), but they can be hypomethylated in cancer cells (Issa, 2004). Hypomethylated repetitive elements have been associated with a high risk of cancer as well as with a decline in organ function (Bollati *et al*., 2009). Thus, hypomethylation of repetitive elements may reflect the degenerative phenotype of aged cells or tissues. Therefore, we compared mean RMS values of LINE, SINE, LTR, simple repeat, and low‐complexity regions of the OLD, OCR, and OCR‐O profiles. The RMS values of these five repetitive elements were decreased in old and OCR rats when compared with young rats, although the RMS values of SIN and LTR elements were slightly increased in OCR rats when compared with old rats (Fig. 2B). The biological mechanism of LINE element hypomethylation by short‐term CR is currently unclear, and further investigation is required. In the OCR‐O profile (DMRs for OCR vs. old rats), the RMS values of SINE and LTR in OCR rats were higher and were lower in LINE, simple repeat, and low‐complexity regions than those of old rats (Fig. 2B).

To evaluate methylation changes in gene body regions, we compared each mean RMS value of the 5′‐UTR, promoter (−1.5K to + 0.5K from transcription start site), exon, intron, and 3′‐UTR in the OLD, OCR, and OCR‐O profiles (Fig. 2C). Results show, interestingly, opposite methylation patterns, unlikely CGI or repetitive elements. For example, methylation on 5′‐UTR, exon, and 3′‐UTR in old and OCR rats was higher than in young rats. Methylation levels in the promoter and intron regions were decreased with age; however, they were increased in OCR rats compared to young rats. The OCR‐O profile shows the RMS values of the five gene body regions to be higher in OCR rats than those of old rats (Fig. 2C).

### Pathway enrichment analysis for differently methylated promoters with twofold change

**Figure 3:**
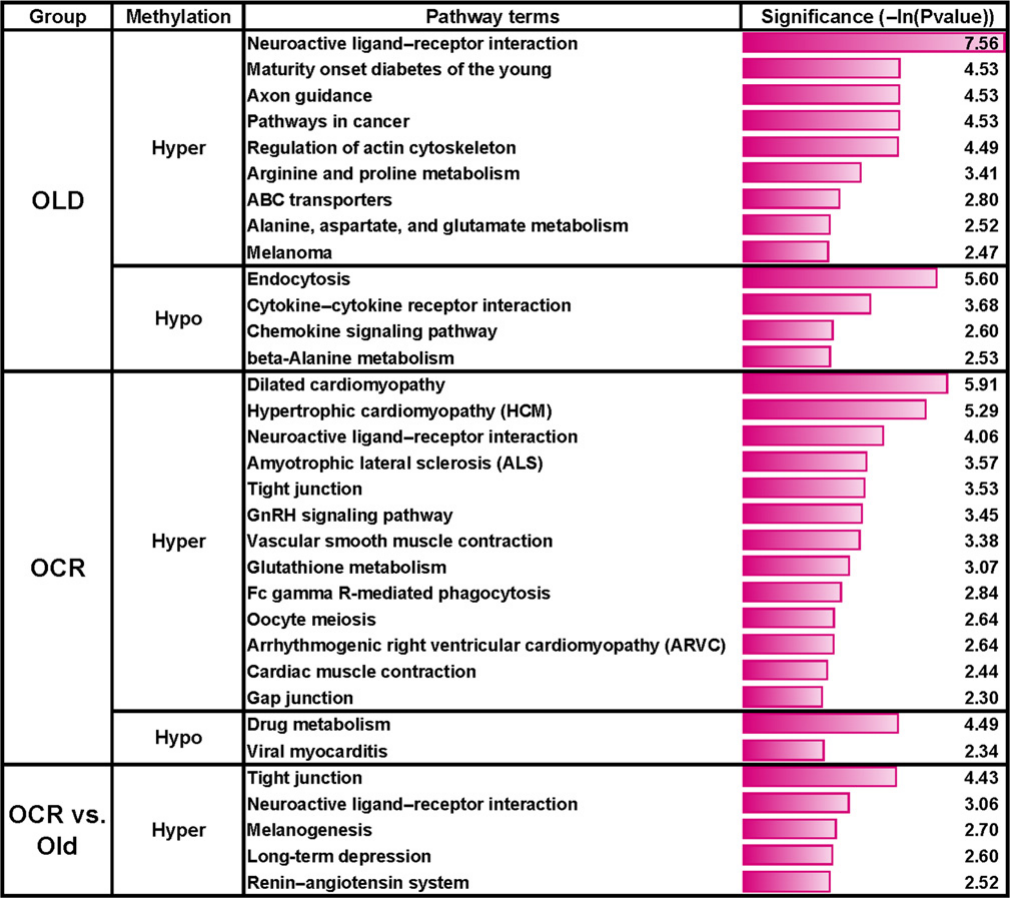
Significant signal pathways of differently methylated promoters in the OLD, OCR, and OCR‐O profiles. The number on each bar indicates the significance value as –Ln(Pvalue).

We performed KEGG pathway enrichment analysis in the hypermethylated and hypomethylated promoter regions that have a significant twofold change. For this, the promoter region was defined as the region between 1.5‐kb upstream and 0.5‐kb downstream from the transcription start site. In the OLD profile, 633 hypermethylated and 368 hypomethylated promoters were identified (Table S6). Notably, both hypermethylated and hypomethylated promoters were significantly enriched in the age‐related phenotypes (Fig. 3). For example, hypermethylated promoters were associated with pathways such as neuroactive ligand–receptor interaction, cancer, diabetes, and nitrogen metabolic process (arginine and proline metabolism, alanine, aspartate, and glutamate metabolism). In contrast, hypomethylated promoters were highly associated with cytokine–cytokine receptor interaction and chemokine singling pathways, which are involved in the inflammation response (Fig. 3 and Table S9).

In the OCR profile, 854 hypermethylated and 327 hypomethylated promoters were identified (Table S7). Interestingly, hypermethylated promoters were enriched in the cardiomyopathy or cardiac muscle contraction, Fc gamma R‐mediated phagocytosis, reproduction (GnRH, oocyte meiosis), and glutathione metabolic pathways. On the other hand, hypomethylated promoters were significantly associated with drug metabolism and viral myocarditis (Fig. 3 and Table S9). From the OCR‐O profiles, 477 hypermethylated and 256 hypomethylated promoters were found (Table S8). The hypermethylated promoters were enriched in tight junction, neuroactive ligand–receptor interaction, melanogenesis, long‐term depression, and renin–angiotensin system pathways, while there were no significant pathways associated with hypomethylation (Fig. 3 and Table S9). Among the enriched pathways, the neuroactive ligand–receptor interaction pathway was common among the OLD, OCR, and OCR‐O profiles, and tight junction was shared between the OCR and OCR‐O profiles.

### Validation of DMRs

For DMR validation, the promoter regions of SDC1 (Syndecan1), PRCKA (protein kinase C‐alpha), and RGN (Regucalcin; SMP30) were selected and sequenced with bisulfite conversion (Fig. S3). SDC1 was hypermethylated in both OLD and OCR profiles. Sequenced SDC1 regions contained seven CpG sites that showed a similar ratio between CG and TG, or more TG in young rats (Tables S2 and S3, and Fig. S3A). In contrast, three CpGs among seven sites were methylated in old and OCR rats. PRKCA showed hypermethylation in the OCR‐O profile. Sequencing results showed that six CpGs in PRKCA were methylated in young rats; however, a more methylated pattern was indicated in the old and OCR rats (Table S4 and Fig. S3B). RGN (SMP30) was also hypermethylated in the OLD profile. The sequencing results from the RGN query showed six CpG sites of this gene were absolutely unmethylated in young rats; however, all sites were methylated in old and OCR rats, although CpG sites in OCR rats were slightly demethylated than those of old rats (Table S2 and Fig. S3C).

## Discussion

Aging is a biological process associated with a progressive loss of cell homeostasis and altered gene expression (Knapowski *et al*., 2002). Epigenetic regulation of gene expression involves various chemical modifications to DNA and DNA‐binding proteins. Previous studies have shown that aging correlates strongly with a global reduction in DNA methylation in mouse, rat, and human (Wilson & Jones, 1983; Singhal *et al*., 1987; Wilson *et al*., 1987; Fuke *et al*., 2004), although this view is controversial with regard to global hypomethylation with age (Tawa *et al*., 1992). Existing as a large portion of the genome, transposable repetitive elements such as LINE‐1 and Alu are associated with genome instability, translocation, and gene disruption via transposition or recombination, which are frequently associated with human disease. Recent studies show that demethylation of these elements over time may contribute to human senescence and degenerative diseases. For instance, a cohort study of healthy elderly subjects showed that methylation in LINE‐1 and Alu decreases over time (Bollati *et al*., 2009). Also, reduced methylation of repetitive elements might contribute to a higher risk of developing and dying from cancer (Zhu *et al*., 2011). These findings are consistent with our data showing five repetitive elements in old and OCR rats that were hypomethylated compared with young rats (Fig. 2B). Thus, the aberrant methylation of repetitive sequences may contribute to genomic instability during aging, and it is thought that short‐term CR could not prevent the demethylation process. However, differential methylation may occur in specific regions of the genome. For instance, a recent pyrosequencing study of human tissues showed that, with age, methylation decreased at loci outside of CpG islands but increased at loci within CpG islands (Christensen *et al*., 2009). In addition, hypermethylation of CpG islands in cancer‐associated genes was observed in 45 different normal prostate tissues (Kwabi‐Addo *et al*., 2007).

Interestingly, although genomewide hypomethylation occurs with aging, gene body regions showed different methylation patterns among the groups studied. Except for the promoter and intron regions, the UTRs and exon regions were highly methylated in the old and OCR groups. Methylation in the promoter and intron regions decreased with age and was ameliorated in OCR rats (Fig. 2C). Although genomewide hypomethylation occurs with aging, the promoter regions of many genes tend to switch from unmethylated to methylated status, resulting in gene silencing (Issa *et al*., 2001; Waki *et al*., 2003). Such a change may occur in the promoters of tumor‐ and age‐related genes. For example, methylation of RASSF1A, a tumor suppressor gene, gradually increases with age. Methylation of RASSF1A is a risk factor in adiposity, renal cancer, and breast cancer (Peters *et al*., 2007; Euhus *et al*., 2008). Alzheimer's disease is an age‐related neurological disease. In Alzheimer's patients, the methylation level of the longevity‐related gene, HTERT, was higher than that of the elderly controls (Silva *et al*., 2008).

Short‐term CR is shown to improve disease‐related markers in old male monkeys (Lane *et al*., 2000), affect age‐related gene expression patterns (Cao *et al*., 2001), attenuate inflammatory signals mediated by oxidative stress (Jung *et al*., 2009a), reduce oxidative damage, and increase the expression of the longevity genes SIRT1 and AMPK (Ning *et al*., 2013). The positive physiological effects from short‐term CR on aging may be partly accompanied by methylation regulation from short‐term CR. Our signal pathway analysis of hypermethylated promoters in old rats showed a significant association with degenerative diseases such as cancer and diabetes; the hypomethylated promoters were related to inflammation (Fig. 3).

**Figure 4:**
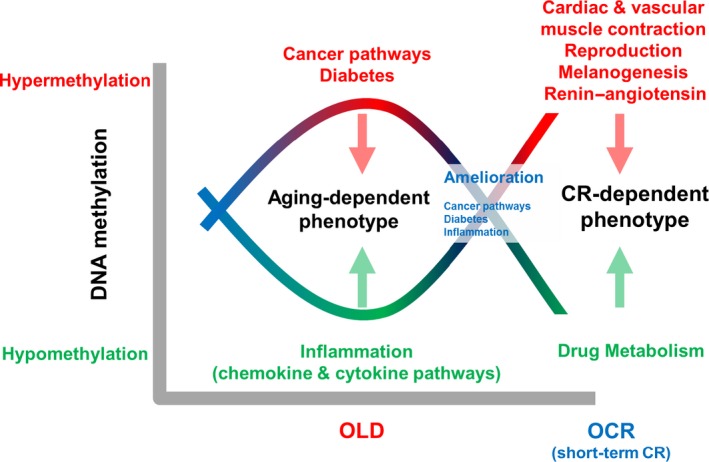
Summarized explanation of methylome changes with age and the short‐term CR effect. Hypermethylated biological processes are indicated with red letters, and hypomethylated biological processes are indicated with green letters.

These results support the our previous findings such as age‐related increases in oxidative stress and a chronic micro‐inflammatory response (Yu & Yang, 1996; Chung *et al*., 2001, 2002; Yu & Chung, 2001). Also, current findings suggest that age‐related, aberrant methylation could increase susceptibility for developing degenerative diseases. In addition, the enriched pathways such as cancer, diabetes, and inflammation in the OLD profile were not observed in the OCR‐O profiles. For instance, comparisons of OCR rats with old rats showed different pathways such as cardiomyopathy (or cardiac muscle contraction), reproduction, and glutathione metabolism (Fig. 3). These results indicate the promoters that were hypermethylated in old rats were ameliorated or hypomethylated by short‐term CR. Venn diagram analysis of DMRs also supports this finding (Fig. 1). Thus, the methylation of these genes occurring at an early phase of calorie restriction may contribute to the CR‐dependent phenotypes, such as longer lifespan (Fig. 4). Our data show that short‐term CR could regulate age‐dependent methylation patterns throughout the genome in a gene‐specific manner. Recent studies suggest that CR reverses aging‐dependent DNA methylation at specific loci involved in oncogenesis and genomic stability (Vaquero & Reinberg, 2009; Munoz‐Najar & Sedivy, 2011). For example, methylation of the proto‐oncogene RAS was higher in CR rats than in rats fed *ad libitum* (Hass *et al*., 1993). Hypermethylated promoters are recognized by transcriptional repressors, which would silence an oncogene and contribute to the cancer‐preventative effects of CR. For example, the tumor suppressor genes CDKN2A (p16^INK4a^) and *TP53* are downregulated by CR via promoter methylation and histone acetylation (Li *et al*., 2010b).

Few reports have described the regulation of methylation by short‐term CR. CR is the most consistent way to increase lifespan and delay the onset of diverse aging‐associated diseases (Fontana & Klein, 2007). CR reverses the decreasing trend in methylation that occurs with aging, an effect associated with increased activity of DNA methyltransferase 1 (DNMT1) (Li *et al*., 2011). CR also changes DNMT3A levels in the mouse brain, suggesting that changes in methylation are associated with the effects of CR on brain function (Chouliaras *et al*., 2011). In human stem cells, DNMT genes play a critical role in the regulation of cellular senescence (So *et al*., 2011). Thus, CR may change the expression and activity of DNMTs, thereby altering DNA methylation. Although these phenomena are observed in long‐term CR studies, indeed CR's effects may happen at an initial phase of long‐term CR (i.e., short‐term CR). CR results in energy and nutrient depletions that lead to dramatic physiology and biochemical changes in single cells as well as organisms. However, to elucidate the molecular mechanism that regulates the whole‐genome methylation process during aging and short‐term CR (and/or long‐term CR) requires further studies. The epigenetic regulation of proteins also can modulate the effects of CR. The NAD+‐dependent protein deacetylase SIRT may be linked to aging and CR. SIRT1 expression and activity decrease with age, but increase with CR (Marton *et al*., 2010; Kitada *et al*., 2013). SIRT modulation by CR also affects histone acetylation. Histone acetylation is regulated by acetyltransferases and histone deacetylases (HDACs). The acetylation levels of lysines 9, 27, and 56 in histone H3 and lysine 16 in H4 were found 30% higher in CR rats than in rats fed *ad libitum* (Kawakami *et al*., 2012). These data suggest that the effect of CR on lifespan may also involve CR‐modulated epigenetic regulation of HDACs and histones.

In this current study, kidney tissue was selected for investigation based on its sensitivity to redox change, high metabolic activity, inflammatory responsiveness, and its proven suitability for aging studies (Jung *et al*., 2009b). Although the selection sites of the upper cortex regions of rat kidney tissues were carefully performed, we could not exclude the possibility of including some degree of noncortex tissue. Hence, this technical problem might impose some limitation on the interpretation of our results.

In summary, we have performed a comparative and comprehensive genomewide DNA methylation analysis of young, old, and OCR (old with short‐term CR) rats to investigate age‐related methylation and the effects of short‐term CR on age‐related DNA methylation. We found the DMRs in old and old with short‐term CR rats, and pathway enrichment analysis of promoter regions in the age‐related DRMs showed that the age‐related alterations in DNA methylations significantly affected the genes involved in the age‐related degenerative diseases. However, those kinds of pathways were not observed from the DMRs in the OCR rats. Our results suggest that short‐term CR could partially ameliorate age‐related methylation changes in promoters in old rats. Therefore, we propose that the aberrant methylation found in the promoter regions of disease‐related genes during aging may indicate increases in susceptibility to age‐related diseases. The CR‐induced methylation changes that ameliorate age‐dependent aberrant methylation may be important for the CR effects such as the health‐ and life‐prolonging phenotypes.

## Material and methods

### Animals

All animal experiments were approved by Pusan National University Small Animal Care and Use Committee. Male SPF Sprague‐Dawley rats were used in this study at 6 months (young rats) and 25 months (old rats) of age. The effects of a 4‐week, short‐term CR regimen were assessed in 25‐month‐old rats (OCR rats). Each group included six rats. Procedures for the maintenance of specific pathogen‐free (SPF) conditions and the dietary composition of chow were previously reported (Yu *et al*., 1985). Briefly, rats were fed a diet containing 21% soybean protein, 15% sucrose, 43.64% dextrin, 10% corn oil, 0.15% α‐methionine, 0.2% choline chloride, 5% salt mix, 2% vitamin mix, and 3% Solka‐Floc fiber. The diet was prepared by the Purina Test Diet Lab (St. Louis, MO, USA). Young and old rats had free access to food and water. The OCR rats were fed 60% of the amount of food consumed by old rats for 1 month beginning at 24 months. The upper cortex regions of rat kidney tissues were used to extract genomic DNA. Rats were sacrificed by decapitation after anesthesia, and the kidneys were quickly removed and immediately frozen in liquid nitrogen and stored at −80 °C.

### Methylated DNA immunoprecipitation, sequencing, and data processing

Rat kidney gDNA was purified using a DNeasy Tissue kit (Qiagen, Hilden, Germany) according to manufacturer's instructions. A DNA sequencing library was prepared from 5 μg of rat kidney gDNA, and the DNA was fragmented with a Covaris S‐2 ultrasonicator (Covaris, Woburn, MA, USA). End repair, A‐base addition, and adaptor ligation steps were performed using Illumina's Paired‐End DNA Sample Prep kit (Illumina, San Diego, CA, USA) according to the manufacturer's instructions. Adaptor‐ligated DNA was immunoprecipitated by anti‐5mC antibody (Li *et al*., 2010a), and MeDIP products were validated by qPCR using SYBR Green Master Mix (Life Technologies, Grand Island, NY, USA); the primers for positive and negative control regions were supplied in the MeDIP kit (Diagenode, Seraing, Belgium). The qPCR program was at 95 °C for 5 min, followed by 40 cycles at 95 °C for 15 sec and 60 °C for 1 min. MeDIP DNA was purified with a ZYMO DNA Clean & Concentrator‐5 column according to the manufacturer's instructions and amplified by adaptor‐mediated PCR. Amplified DNA between 220 and 320 bp was excised from a 2% agarose gel and purified using a QIAquick Gel Extraction Kit (Qiagen). Amplification quality and quantity were evaluated with an Agilent 2100 Analyzer and DNA 1000 chips (Agilent, Santa Clara, CA, USA). The library was quantified against a PhiX control library by qPCR using a LightCycler 480 (Roche, Mannheim, Deutschland) according to the library quantification protocol provided by Illumina. DNA was sequenced with 50‐bp paired‐end reads and a HiSeq Sequencing Kit using the Illumina HiSeq 2000 according to the manufacturer's instructions. The raw fluorescent images and call sequences were processed with a base‐calling pipeline (Sequencing Control Software ver. 1.7). After Bowtie2 mapping (Langmead & Salzberg, 2012) of the MeDIP‐seq reads against the rat reference genome rn5 build downloaded from UCSC (http://genome.ucsc.edu) obtained approximately 96 million unique high‐quality (MAQ > 20) mapping hits for young, old, and OCR rats.

### Identification of DMRs and analysis of signal pathways

To detect DMRs, we followed the basic MEDIPS protocol described by Chavez *et al*. (Chavez *et al*., 2010). Using the MeDIP‐seq data from young, old, and OCR rats, respectively, we calculated the short‐read coverage (extend value = 500) at genomewide 50‐bp bins using MEDIPS. To identify DMRs, MEDIPS calculates the mean RPKM (for each group) and the mean relative methylation score (rms) values for overlapping genomewide 500‐bp windows where neighboring windows overlap by 250 bp. Additionally, MEDIPS calculates *P*‐values by comparing the rms signal distributions of the 50‐bp bins of young, old, and OCR groups within each of the 500‐bp windows. Significant DMRs were identified by filtering for windows associated with a Poisson's test *P*‐value ≤ 0.05, a mean RPKM exp/control ratio ≥ 1.33 for hypermethylation, or a mean RPKM exp/control ratio ≤ 0.75, which were calculated by EdgeR algorithm in MEDIPS program (Nikolayeva & Robinson, 2014). RMS values of significant DMRs were used for methylation analyses of repeat elements and gene body regions. For pathway enrichment, promoters were defined as the DMR within −1.5K to +0.5K from the transcription start site and with a significant, twofold methylation change. Annotation of promoter regions was performed using Homer (Hypergeometric Optimization of Motif EnRichment) software (Heinz *et al*., 2010). Signal pathway analyses were performed on the sets of unique Entrez gene identifiers of promoters using the DAVID Bioinformatics Resources 6.7 Functional Annotation Tool (Huang da *et al*., 2009). Gene sets were identified by joining subsets of the MeDIP‐enriched regions with RefSeq tables obtained from the UCSC tables.

### Validation of DMRs with bisulfite sequencing

DMR validation was performed via bisulfite sequencing. Chromosomal positions of the DMRs in promoter regions SDC1, PRKCA, and RGN (SMP30) were obtained from MEDIPS data, and then, the sequence of MDR ± 1.5 Kbp was downloaded from the UCSC. From this sequence, a bisulfite sequencing primer was designed using MethPrimer tools (http://www.urogene.org/methprimer/); the oligo sequences are provided in Table S9.

Same amounts of gDNA of three rats from each group were pooled and conversed using the EZ DNA methylation Gold Kit (Zymo Research, CA, USA) according to manufacturer's instructions. Bisulfite conversion condition was 10 min at 98 °C and 2.5 hr at 64 °C. The conversed DNA was amplified with the bisulfite sequencing primer and used for Sanger sequencing.

## Funding

This work was supported by the National Research Foundation of Korea (NRF) grant funded by the Korea government (MSIP) (No. 2009‐0083538) and the Reference genomes building and application for large scale population genomics Research Fund (1.160003.01) of UNIST (Ulsan National Institute of Science & Technology).

## Conflict of interest

There is no potential conflict of interest to disclose.

## Supporting information


**Fig. S1** Summary of alignment of MeDIP‐Seq reads to reference rats genomes, for young (A), old (B) and OCR (C). MAPQ; Mapping and Assembly with Qualities, OCR; old rats with caloric restriction.
**Fig. S2** Comparison design for methylome analysis.
**Fig. S3** Chromatogram of bisulfite sequencing of DMRs of SDC1 (A), PRKCA (B), and RGN (C).

**Table S1** Summary of sequencing output. A sequence read is single‐ended 49 bp (unique >= MAQ20).

**Table S2** Full DMR profile from old vs. young rats.
**Table S3** Full DMR profile from OCR vs. young rats.
**Table S4** Full DMR profile from OCR vs. old rats.

**Table S5** The profile of promoters with short‐term, CR‐mediated amelioration of age‐related, aberrant methylation.

**Table S6** Hypermethylated or hypomethylated promoter regions from old vs. young rats.

**Table S7** Hypermethylated or hypomethylated promoter regions OCR vs. young rats.

**Table S8** Hypermethylated or hypomethylated promoter regions from OCR vs. old rats.

**Table S9** Pathway enrichment results of differentially methylated promoter regions.

**Table S10** Bisulfite sequencing primers for DMR validation.
