# Supplementary material for: Short‐term calorie restriction ameliorates genomewide, age‐related alterations in DNA methylation
Source: Aging Cell. 2016 Aug 25;15(6):1074–81. doi: 10.1111/acel.12513 (PMC6398531; doi:10.1111/acel.12513)
Supplement: Supplementary file 2 — Table S1 Summary of sequencing output. A sequence read is single‐ended 49 bp (unique >= MAQ20). [file ACEL-15-1074-s002.docx]

Supplementary Table S1. Summary of sequencing output. A sequence read is single-ended 49 bp (unique >= MAQ20)

| Sample | Total Clean  Reads (M) | Data Size (Gbp) | Q-Score (mean) | Mapped Reads (M) | Total Mapping  Rate (%) | Unique Mapped Reads (M) | Unique Mapping Rate (%) |
| --- | --- | --- | --- | --- | --- | --- | --- |
| Young-1 | 22.8 | 1.11 | 38.69 | 21.9 | 96 | 16 | 69.9 |
| Young-2 | 22.8 | 1.11 | 38.67 | 21.9 | 96 | 16 | 70.8 |
| Young-3 | 22.9 | 1.12 | 38.70 | 22.1 | 96 | 16 | 70.1 |
| Young-4 | 22.8 | 1.11 | 38.70 | 22 | 96 | 16.1 | 70.7 |
| Young-5 | 22.8 | 1.11 | 38.70 | 22 | 96 | 16.1 | 70.6 |
| Young-6 | 22.7 | 1.12 | 38.76 | 21.6 | 96 | 16 | 70.5 |
| **Young** | **136.8** | **6.7** | **38.70** | **131.5** | **96** | **96.2** | **70.4** |
| Old-1 | 22.9 | 1.12 | 38.68 | 22 | 96 | 15.7 | 69 |
| Old-2 | 22.9 | 1.12 | 38.70 | 22.1 | 96 | 16 | 70 |
| Old-3 | 22.9 | 1.12 | 38.73 | 22.1 | 96 | 16.1 | 70.8 |
| Old-4 | 22.9 | 1.12 | 38.72 | 22.1 | 96 | 16 | 70.2 |
| Old-5 | 22.6 | 1.1 | 38.17 | 21.7 | 96 | 15.8 | 70.1 |
| Old-6 | 23 | 1.12 | 38.22 | 22.1 | 96 | 16 | 69.9 |
| **Old** | **137.2** | **6.72** | **38.5** | **132.1** | **96** | **95.6** | **70** |
| OCR-1 | 22.7 | 1.11 | 38.28 | 21.9 | 96 | 16.4 | 73.2 |
| OCR-2 | 22.8 | 1.11 | 38,68 | 21.9 | 96 | 15.7 | 69.4 |
| OCR-3 | 22.5 | 1.1 | 38,74 | 21.7 | 96 | 16 | 71.2 |
| OCR-4 | 22.6 | 1.1 | 38.77 | 21.8 | 96 | 16.1 | 71.3 |
| OCR-5 | 22.8 | 1.11 | 38.76 | 22 | 96 | 15.9 | 70.2 |
| OCR-6 | 22.9 | 1.12 | 38.8 | 22 | 96 | 15.9 | 69.7 |
| **OCR** | **136.3** | **6.68** | **25.8** | **131.3** | **96** | **96** | **70.8** |
