# Supplementary material for: Short‐term calorie restriction ameliorates genomewide, age‐related alterations in DNA methylation
Source: Aging Cell. 2016 Aug 25;15(6):1074–81. doi: 10.1111/acel.12513 (PMC6398531; doi:10.1111/acel.12513)
Supplement: Supplementary file 3 — Table S2 Full DMR profile from old vs. young rats. Table S3 Full DMR profile from OCR vs. young rats. Table S4 Full DMR profile from OCR vs. old rats. [file ACEL-15-1074-s003.docx]

The following link is my Google Drive links that contains supplementary tables 2-4.

<https://drive.google.com/folderview?id=0By088vFyZXA6X3hzUlpuQm5nclU&usp=sharing>
