# Supplementary material for: Short‐term calorie restriction ameliorates genomewide, age‐related alterations in DNA methylation
Source: Aging Cell. 2016 Aug 25;15(6):1074–81. doi: 10.1111/acel.12513 (PMC6398531; doi:10.1111/acel.12513)

Supplementary Figure S1.

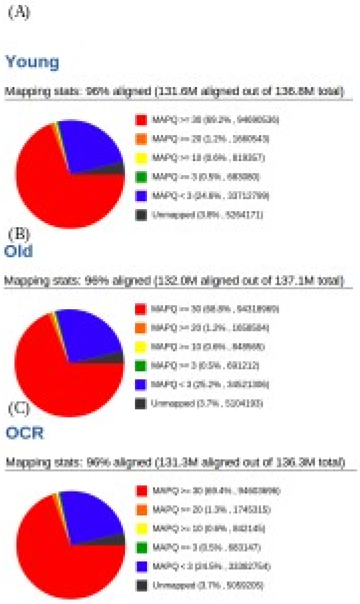

Supplementary Figure S2.

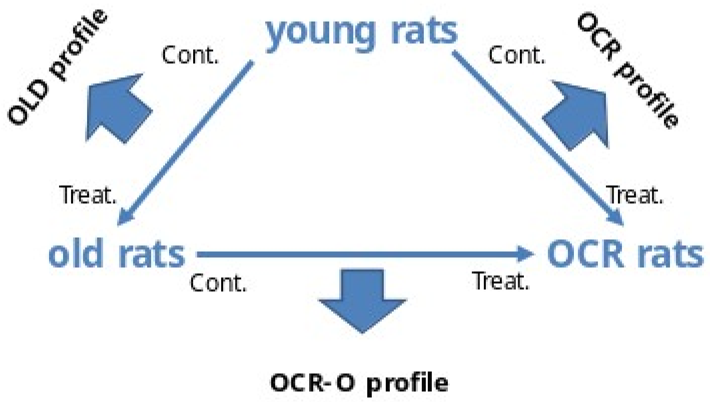

Supplementary Figure S3A

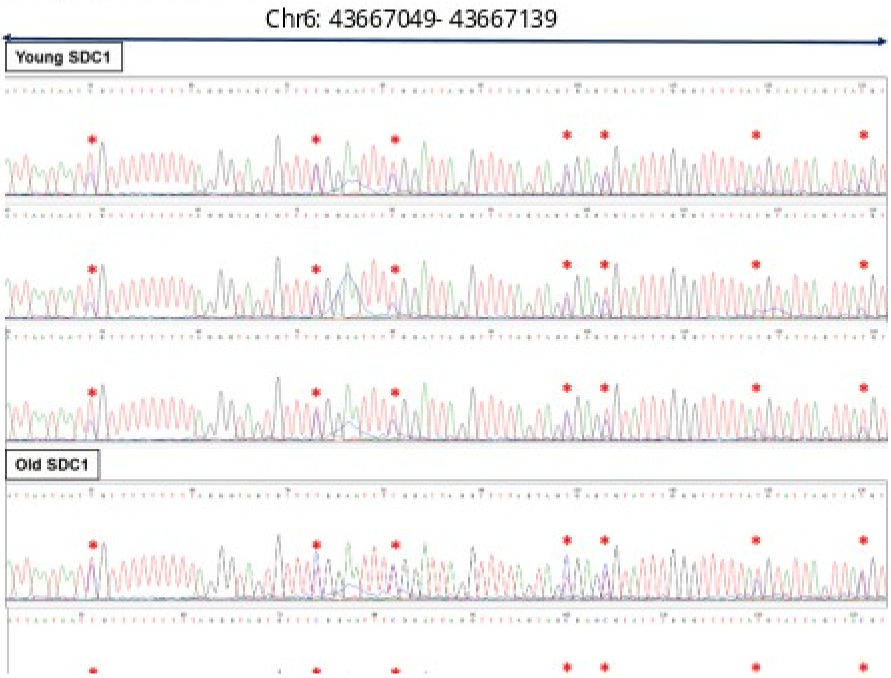

Supplementary Figure S3B

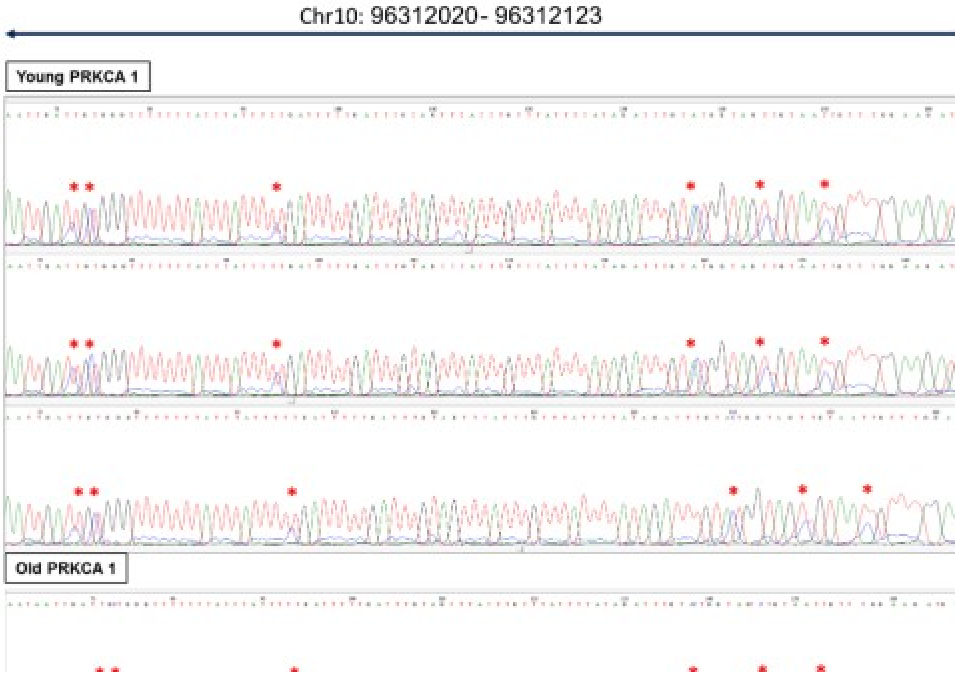

Supplementary Figure S3C

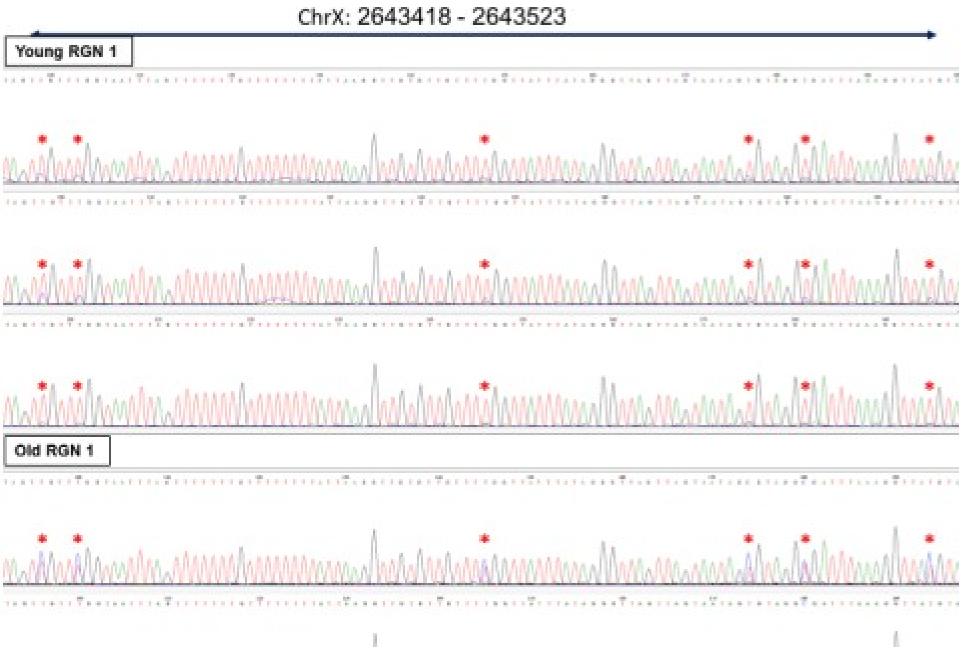

Supplement: Supplementary file 3 [file ACEL-15-1074-s001.docx]
